# Applying a hexapod circular frame to gradually correct persistent knee flexion due to congenital aplasia of the quadriceps—a case report and review of the literature

**DOI:** 10.3389/fped.2025.1631106

**Published:** 2025-10-17

**Authors:** Maxime Pilloux, Victor Aye, Dimitri Fasel, Elio Paris, Giacomo De Marco, Oscar Vazquez, Christina Steiger, Romain Dayer, Sana Boudabbous, Dimitri Ceroni

**Affiliations:** ^1^ Faculty of Medicine, University of Geneva, Geneva, Switzerland; ^2^ Paediatric Orthopaedics Unit, Paediatric Surgery Service, Geneva University Hospitals, Geneva, Switzerland; ^3^ Radiology Department, Geneva University Hospitals, Geneva, Switzerland

**Keywords:** persistent knee flexion, congenital aplasia, quadriceps, pediatric, hexapod circular frame

## Abstract

Congenital aplasia or hypoplasia of the quadriceps muscle are rare conditions that significantly impair the extension of the knee joint, resulting in a sustained flexion deformity. These conditions alter overall gait quality, impede daily activities and require effective, timely intervention. Surgical techniques for correction have been described, including soft-tissue release, guided growth, tendon transfer and external fixators. Among the external fixators available, hexapod circular frames are probably the most effective because they enable the precise, progressive correction of complex multiaxial deformities. We present the case of a 10-year-old girl with quadriceps aplasia and a severe flexion contracture of the knee. Surgical treatment involved the mobilisation of the patella, a transfer of hamstring tendons and the application of a hexapod circular frame to control the correction of the flexion deformity. This case study provides a glimpse into the use of hexapod circular frames to remediate severe knee flexion deformities that were previously believed to be irreversible due to the patient's quadriceps muscle aplasia. Safe, controlled aplasia correction greatly improves children's gait and functional ability to move around, making it an invaluable technique for managing difficult congenital knee problems.

## 1 Introduction

Congenital aplasia or hypoplasia of the quadriceps remain very rare conditions, but they significantly impair the extensor mechanisms of the knee. Congenital aplasia was first described by Drachmann in 1873 (1). Since then, only 22 similar cases have been described with reporting on diagnostic testing and therapeutic management (2–7). Congenital quadriceps abnormalities reveal themselves early on during organogenesis, at the 5th week for aplasia and between the 10th and 16th weeks for hypoplasia (7). Aplasia and hypoplasia of the quadriceps can both be the result of an overall context of malformation and are sometimes associated with the congenital absence of a patella (2–6). The main clinical manifestations of aplasia or hypoplasia of the quadriceps involve an absence of the ability to extend the leg and persistent knee flexion (7). Some authors have distinguished three clinical types according to their anatomical characteristics and functional implications. In complete aplasia, all four heads of the quadriceps muscle are absent, with only fat present in their place. Usually, only one limb is affected, but any active extension of the knee is impossible. Continuous hypoplasia is characterised by the unilateral hypoplasia of the four prevailing muscles making up the quadriceps, often combined with hypoplasia of the femur and tibia, as well as knee joint abnormalities. In cases like this, however, active extension of the knee is generally feasible. Finally, cases of discontinuous hypoplasia involve hypoplasia of just some of the muscle heads, usually the rectus femoris and the vastus intermedius, and cases of aplasia usually involve just the distal third of the quadriceps. In aplasia, active knee extension is impossible or severely impaired (7). Without treatment, patients with aplasia or hypoplasia of the quadriceps will be unable to or have extreme difficulty extending the knee and will experience continued major functional difficulties during walking. The successful management of such complex deformities relies on precise preoperative and postoperative assessment, including the knee joint function and symmetry in both the coronal and sagittal planes to quantify the deformity (8, 9).

The purpose of this case report was to describe a simple and more modular surgical technique aimed at correcting a flexion contracture of the knee. The novelty of this case is the demonstration, for the first time, that this method can achieve a functional correction in a condition previously considered untreatable with external fixation.

## 2 Case report

**Figure 1:**
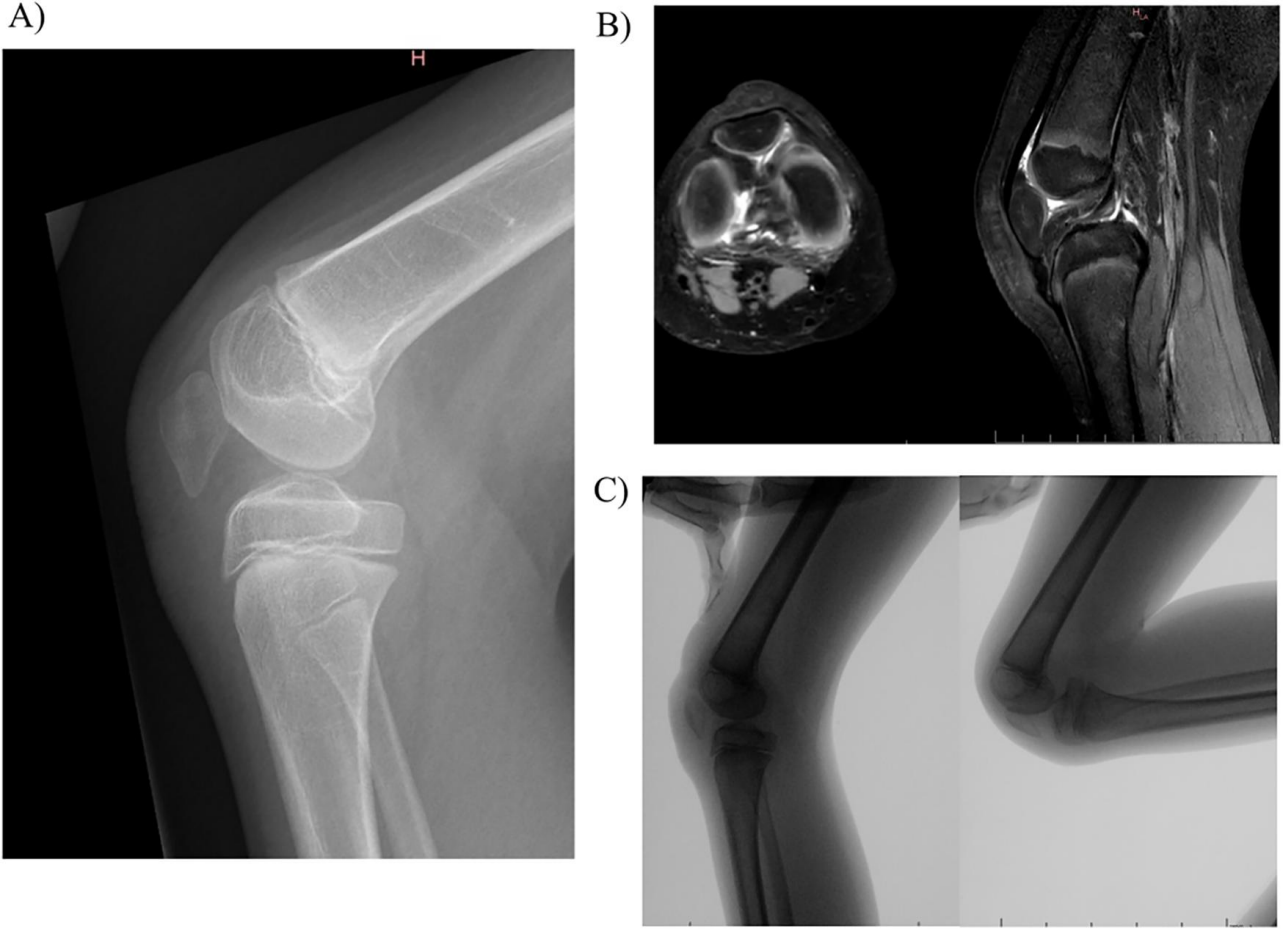
Baseline evaluation of the knee: **(A)** conventional radiography; **(B)** magnetic resonance imaging; **(C)** dynamic radiological examination.

A ten-year-old girl was referred to our paediatric orthopaedics unit for aplasia of the right quadriceps accompanied by a persistent knee flexion deformity. The diagnosis had been made at the child's birth. She had initially been treated at another hospital, and she had undergone initial surgery at six years old. This consisted of an anterior distal femoral hemi-epiphysiodesis (with guided growth), which managed to restore complete knee extension. Over the following four years, the knee flexion deformity gradually returned, significantly hindering the walking process. Upon initial clinical examination, the anterior part of her thigh was observed to be severely atrophied, and the knee's flexion deformity was noted to have an extension defect of 40° but with no popliteal pterygium contracture. She was able to bend the knee to 120°. A conventional radiograph revealed an abnormally low-lying patella (Figure 1A), which appeared to be embedded in the femoral notch; it also showed a posterior static drawer due to a muscular disbalance between the knee flexors and extensors. However, no deformity of the distal femur or the proximal tibia was noted. Magnetic resonance imaging revealed that the patella was indeed stuck in the notch and that its shape had adapted to the local mechanical constraints (Figure 1B). The quadriceps muscle was severely aplastic, and although a quadricipital tendon was present, it was poorly developed and perpetuated proximally by a fibrous remnant. A dynamic radiological examination was carried out and demonstrated that passive extension was hindered by the position of the patella, which impinged between the femur and the tibia (Figure 1C).

**Figure 2:**
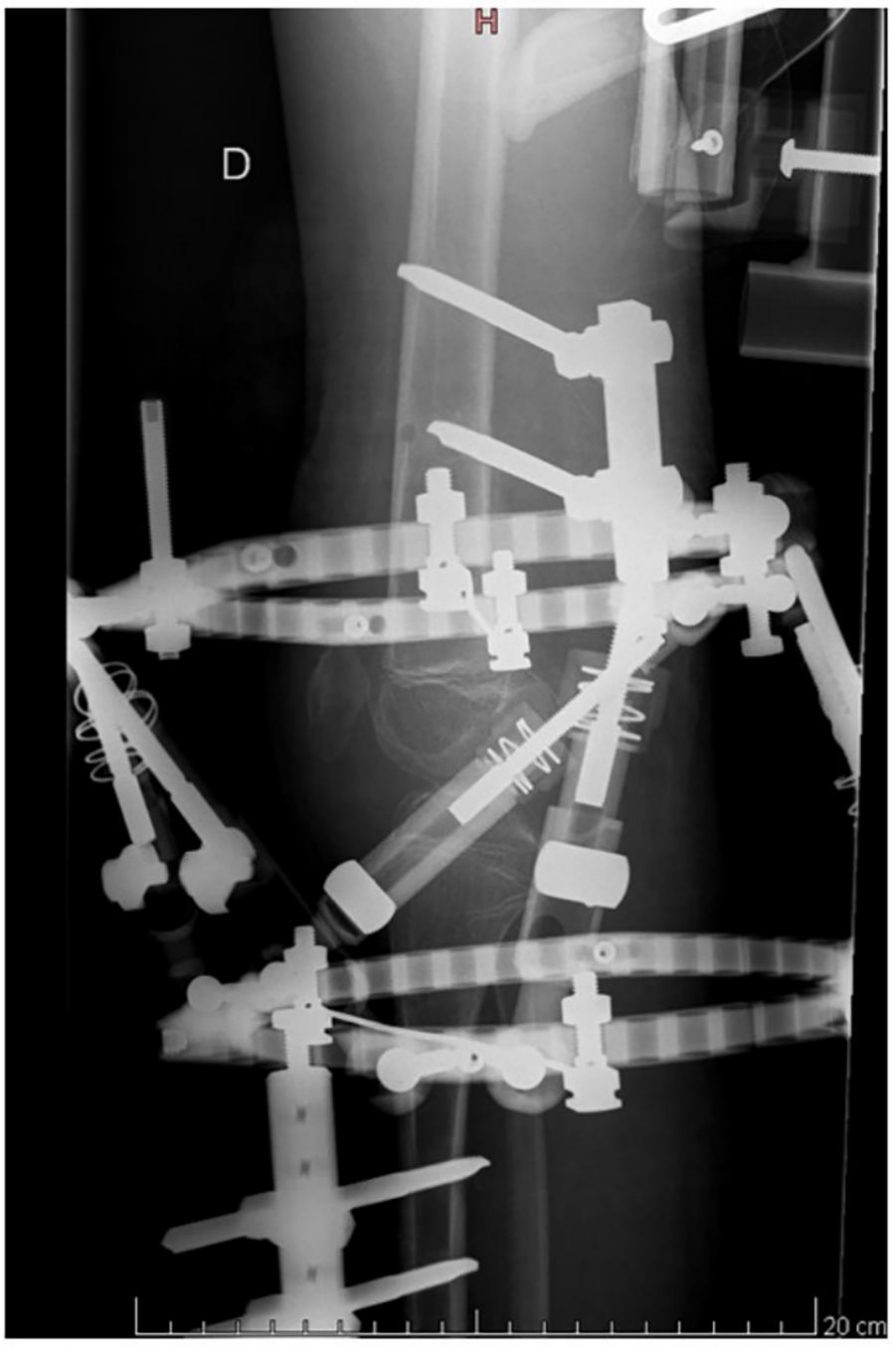
Radiological evaluation of the knee after correction with hexapod circular frame.

An arthroscopic investigation could not be performed during surgery due to a complete obliteration of the anterior compartment of the knee. A medial arthrotomy of the knee was performed, which confirmed that the patella was stuck in the notch and kept in position by an abundant scarring reaction, literally glueing the patella to the anterior cruciate ligament (ACL). The patella was then freed from the notch, and the tendons of the biceps cruralis and semitendinosus muscles were loosened from their distal insertions and transferred to the patella's proximal pole. Given that the deformation had a three-dimensional component, we selected the hexapod circular external fixator as the optimal therapeutic solution. This system is both simpler to use and provides more predictable outcomes. This fixator was applied to gradually correct the patient's knee flexion, beginning with the correction of the proximal tibia's posterior translation. Complete correction of the knee flexion deformity and the posterior static drawer was obtained in just 18 days (Figure 2). Safety of progressive correction was ensured by ensuring that it does not result in an elongation of the vascular-nervous structures greater than 1 mm per day. The external frame device was left in place for a further 21 days and then removed. After removal of the external fixator, a static extension orthosis was used to minimize tension on the tendon transfer. Rehabilitation then focused on regaining active range of motion, by reactivating the transferred tendons. The patient was trained to use former function of the transferred tendon and the new function simultaneously, initially by mirroring movements with the contralateral limb, and then performing exercises bilaterally.

**Figure 3:**
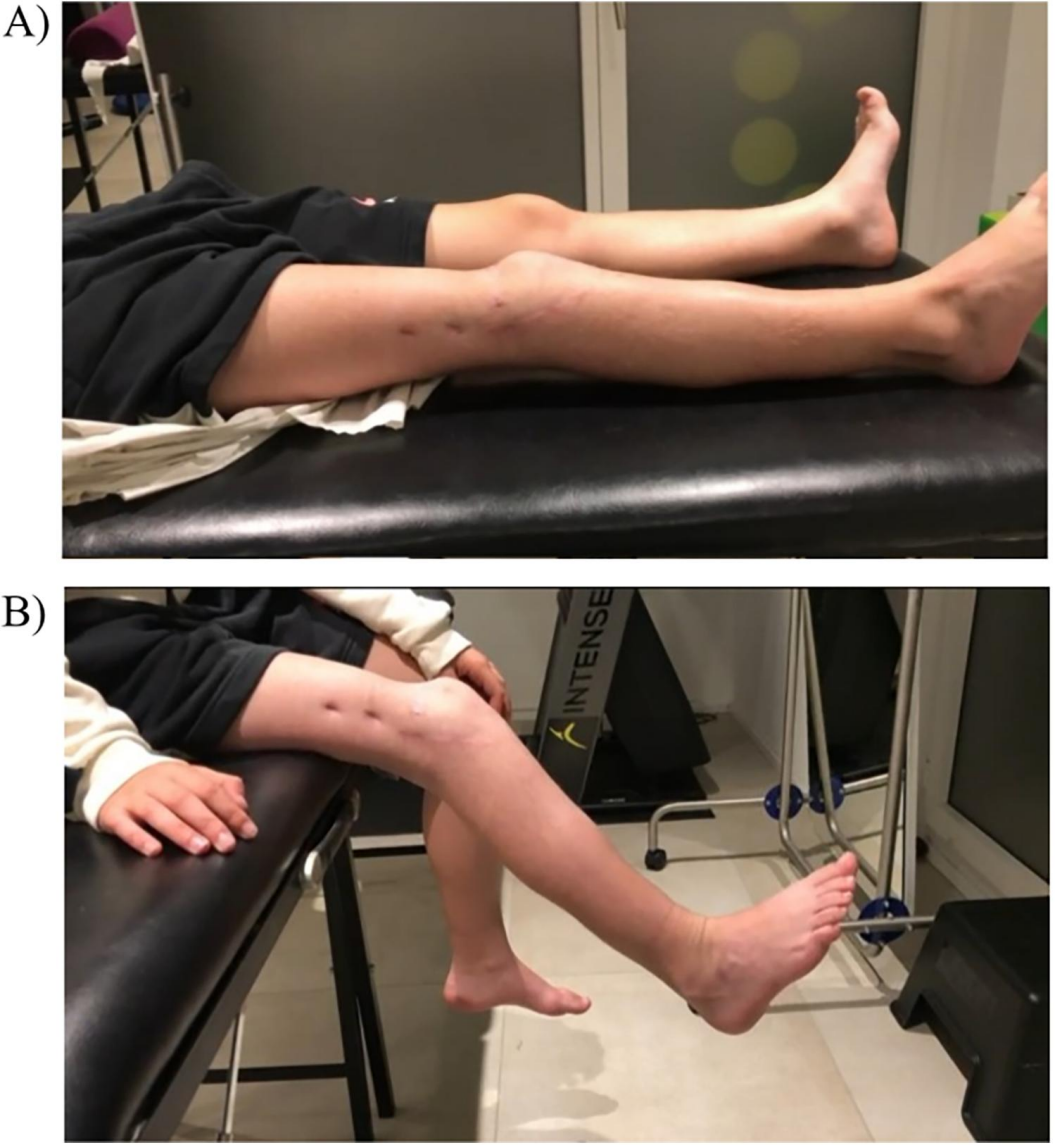
7-month follow-up clinical evaluation with: **(A)** knee extension complete and active flexion at 40°; **(B)** graded 3 in a knee extension resistance test.

At the 11-month follow-up, knee extension was complete, and active flexion was at 60° (Figure 3A). The patient was graded 3 in a knee extension resistance test and was able to walk without orthosis with only a slight limp (Figure 3B).

## 3 Discussion

This case report aimed to describe the clinical presentation of a unilateral aplasia or hypoplasia of the quadriceps and the different problems that its therapeutic management must address. We also tried to prioritise the specificities that surgery must deal with and to understand the results that one might legitimately expect depending on the surgical procedures performed. There are three main clinical problems during aplasia or hypoplasia, namely the irreducible flexion deformity, the lack of any ability to actively extend the leg and the limb-length discrepancy. It is well-known that a flexion deformity can be complicated by a pterygium (10). In the present case, the deformity was also worsened by an intra-articular cause that has never previously been described. Indeed, we noted that the patella was stuck in the femoral notch and held in this position by an abundant scarring reaction, literally glueing the patella to the ACL. This led, therefore, to a real impingement between the patella and the femoral notch when extending the knee.

The specifications for surgery must, therefore, include several components. First, it seems essential to obtain the knee's full extension and maintain it. Trying to restore muscular balance is surgery's secondary goal, to avoid any recurrence of the deformity.

The first step in the treatment strategy usually consists of non-operative management combining plaster casts, extension orthoses and physical therapy (7). This treatment regimen should be initiated within the first weeks of life to prevent or at least limit the flexion deformity. However, for severe aplasia or hypoplasia of the quadriceps, non-operative and minimal surgical procedures aimed at preventing a recurrence of flexion deformity are doomed to failure if nothing is done to try to resuscitate the knee extension or at least to decrease the imbalance between the joint's extensors and flexors (7).

Surgery to regain knee extension revolves around bone and capsular procedures. A posterior capsulotomy may be indicated for slight residual flexion deformities. Larger deformities may require bone procedures, such as a corrective distal femoral extension osteotomy or intra-epiphyseal arthrodesis. Some authors have proposed using guided growth via an anterior distal femoral hemi-epiphysiodesis to correct flexion deformities of the knee. Unfortunately, we know that bone remodeling occurs after knee extension osteotomies or anterior hemi-epiphysiodesis, and that the flexion's deformity will recur with the passing of time; it may be therefore necessary to repeat the surgery in the future to regain full extension. Varghese et al. also described tendon transfers to restore knee extension, and the tendons of the semitendinosus and biceps cruralis muscles can be used as grafts and transferred to the rectus femoris tendon, thus allowing us to recreate the quadriceps mechanisms essential for active extension of the knee and bipedal gait. An intra-epiphyseal arthrodesis is usually only considered in the event of a severe knee deformity or when the quadriceps is very weak. However, this procedure should only be performed on teenage patients and as late as possible in their growth process. Some reports have mentioned the use of another approach to the management of congenital flexion deformity of the knee other than aplasia of the quadriceps using Ilizarov's principle of distraction histogenesis (11, 12).

We managed our ten-year-old patient using a method relatively similar to that described by Varghese et al. regarding the surgery's stated goals (5). However, instead of performing a distal femoral extension osteotomy to correct the flexion deformity, we corrected the deformity progressively using a hexapod circular frame. Our choice was made for hexapod circular frame rather than for Ilizarov method, to the extent that this technique offers a greater ability to correct multi-planar deformity in a gradual manner with a much simpler use.

This approach is particularly interesting when dealing with large flexion deformities because it does not require bone shortening, as the Varghese technique does, to achieve soft tissue balance and avoid neurovascular stress (5). It also helps to control the proximal tibia's posterior translation into the sagittal plane. The resuscitation of quadriceps function by transferring the tendons of the semitendinosus and biceps cruralis muscles aims to restore better muscular balance to knee flexion and extension and helps to prevent a recurrence of the flexion deformity.

## 4 Conclusion

This case study highlights the complexity of congenital quadriceps aplasia and reveals factors that can complicate this pathology. In addition to the classic knee flexion contracture due to the absence of knee extension, this case also emphasised that the peculiar intra-articular position of the patella could impinge upon mechanical extension. The deformity was successfully corrected using an external hexapod circular frame, and adequate joint function was restored using a combination of surgical techniques, including patellar mobilisation and tendon transfers. By progressively stretching the tissues, the hexapod frame proved to be an effective alternative to traditional corrective osteotomies, enabling precise correction while reducing the risk of neurovascular complications. The transfer of flexor tendons improves muscle balance around the knee and may prevent a recurrence of knee joint flexion. This case study highlights the importance of using a personalised approach based on a thorough assessment of the muscular and mechanical origins of the patient's deformity. It also validates the growing interest in dynamic corrective approaches to the treatment of challenging congenital orthopaedic pathologies.

## Data Availability

The original contributions presented in the study are included in the article/Supplementary Material, further inquiries can be directed to the corresponding author.
